# Perceived safety and trust shape transgender and gender diverse participation in exercise science: a consensual qualitative research study

**DOI:** 10.3389/fspor.2026.1864737

**Published:** 2026-08-05

**Authors:** Joshua D. Wooldridge, James W. Navalta, Juliet L. Moore, Dustin W. Davis, Jason D. Flatt, Jafra D. Thomas, Lindsey E. Eberman, Whitley J. Stone

**Affiliations:** 1 School of Rehabilitation Sciences, Moravian University, Bethlehem, PA, United States; 2 Department of Kinesiology and Nutrition Sciences, University of Nevada, Las Vegas, Las Vegas, NV, United States; 3 Department of Kinesiology and Applied Physiology, University of Delaware, Newark, DE, United States; 4 Center for Health Disparities Research, Department of Social and Behavioral Health, University of Nevada, Las Vegas, Las Vegas, NV, United States; 5 Department of Kinesiology and Public Health, California Polytechnic State University, San Luis Obispo, CA, United States; 6 Department of Applied Medicine and Rehabilitation, Indiana State University, Terre Haute, IN, United States; 7 School of Kinesiology, Recreation & Sport, Western Kentucky University, Bowling Green, KY, United States

**Keywords:** community engagement, gender minority stress, inclusive research design, participant-centered research, qualitative methods, recruitment strategies, research equity, research participation

## Abstract

**Introduction:**

Transgender and gender diverse people are substantially underrepresented in exercise science research. This limits the inclusivity and generalizability of evidence guiding exercise, physical activity, and physical rehabilitation practices. While prior health research has examined barriers and facilitators to transgender and gender diverse participation, little is known about how transgender and gender diverse individuals perceive exercise science research specifically. The purpose of this qualitative study was to explore factors that may influence transgender and gender diverse individuals' willingness to participate in exercise science research.

**Methods:**

Using a Consensual Qualitative Research approach, we conducted semi-structured interviews with 17 transgender and gender diverse adults recruited via LGBTQIA2S+ university and community organizations in the United States. Data were analyzed via Consensual Qualitative Research methods to identify domains, categories, and subcategories reflecting shared topics and patterns in responses across participants.

**Results:**

Three primary domains emerged: research participation, researcher decisions and behaviors, and safety. Participants described their willingness to participate as influenced by study design features, information clarity, and perceived benefits. Trust in researchers and institutions, inclusive recruitment procedures, and communication were also key facilitators of participation. Barriers included invasive study procedures, exclusionary eligibility criteria, ambiguous recruitment materials, and concerns about gender misclassification or misuse of data. Across domains, perceptions of political, physical, and psychosocial safety served as a central concern through which participants evaluated research opportunities. Participants emphasized that respectful communication, inclusive research practices, privacy protections, and culturally competent researchers were essential for safe and affirming research environments.

**Discussion:**

Our findings highlight the importance of intentional inclusivity in exercise science research design and recruitment. Implementing transparent communication, community-engaged recruitment, inclusive practices, and trauma-informed procedures may improve participation and representation of transgender and gender diverse individuals in exercise science research. Enhancing inclusion may address ethical responsibilities and strengthen the scientific validity and applicability of exercise science evidence.

## 1 Introduction

Transgender and gender diverse (TGD) individuals experience persistent health inequities, including disparities in physical activity participation, access to gender-affirming and general medical care, and inclusion within health research (1–3). A broad body of literature indicates widespread experiences of discrimination, stigma, and subsequent healthcare avoidance among TGD populations (1). These experiences shape TGD individuals' trust in institutions and limit their opportunities for equitable engagement in health-promoting activities and exercise science research. Structural stigma, negative healthcare encounters, and exclusionary systems contribute to worse health outcomes and reduced representation within research samples and datasets for the TGD population (2, 4, 5). These longstanding inequities underscore the need for methodologically rigorous and culturally responsive research practices explicitly inclusive of TGD identities.

Across health research, TGD populations remain substantially underrepresented. Research participation remains limited, even in fields acknowledging the necessity of inclusion, due to exclusionary collection approaches, limited cultural training, and research methods failing to account for gender diversity (2, 6, 7). Previous research investigating underrepresentation highlights fear, lack of trust in researchers, concerns about confidentiality, negative healthcare experiences, and logistical challenges as barriers to research participation (6, 8–10). Optimistically, research has identified facilitators, including affirming environments, transparent communication, community collaboration, and compensation; however, evidence-informed facilitators of participation are inconsistently implemented across studies (8–10). As a result, the research guiding clinical decisions, public health recommendations, and health promotion that impact TGD individuals remains incomplete.

These challenges are amplified within exercise science, including sport as a key subdomain where issues related to participation, identity, and sociopolitical discourse are highly visible (11, 12). While sex-based physiological differences are extensively studied in the field, researchers seldom include TGD individuals as participants unless gender identity is an explicit focus of an investigation (13). Literature in exercise science highlights that nearly all empirical work categorizes participants strictly as male or female, often without clarifying if these categories reflect sex assigned at birth or gender identity, and without acknowledging the existence of TGD individuals (14, 15). This omission creates a systemic barrier to inclusion and makes it difficult to assess whether the lack of participation reflects researcher exclusion, unintentional erasure due to binary measurement frameworks, or self-exclusion by TGD individuals in response to perceived risks of participation or discomfort within exercise research settings.

Meanwhile, TGD individuals often report negative or unsafe experiences in sport and exercise environments. Research examining campus recreation, physical activity, and sport engagement describe gendered spaces, lack of inclusive facilities, and fear of discrimination as barriers to participation (14, 16–18). Although these barriers relate to exercise outside of research contexts, such concerns likely transfer to exercise research. Studies in the field often involve elements that may heighten vulnerability for TGD individuals, such as physical assessments, body exposure, gendered spaces, and close interaction with research personnel. Increased public attention and sociopolitical scrutiny of TGD participation in sport may also influence willingness to participate in exercise research (3, 19, 20). While not the focus of the present study, sociopolitical dynamics form an unavoidable backdrop shaping TGD individuals' perceptions of safety and inclusion. Recent reviews emphasize the need for evidence-based, non-stigmatizing approaches to understanding sports performance and physiology among TGD individuals while acknowledging that political discourse has outpaced scientific evidence (13, 15).

Further highlighting the lack of representation, our previous experiences in secondary analyses of publicly available data found little indication of TGD inclusion in exercise science research (21, 22). Our research observations align with patterns documented in investigations of TGD research participation, where non-inclusive study designs and recruitment materials contribute directly to research disengagement (6, 8–10). This pattern raises critical questions about why TGD individuals are underrepresented in exercise science when gender identity is not a central variable of interest: Are TGD individuals being systematically excluded from participation? Are recruitment strategies failing to reach TGD communities? Or are TGD individuals self-excluding due to perceived risks, discomfort, or unfamiliarity with exercise science? And importantly, what institutional conditions shape whether TGD individuals and communities perceive exercise science research as legitimate and safe?

Beyond limiting representation, systemic exclusion of TGD individuals from exercise science research has broader implications for community segments that interact with exercise science institutions, programs and services. Research investigating TGD perceptions towards exercise science research in terms of participation barriers and benefits will elucidate ways which prevailing norms, practices and policies of sport and exercise science organizations specifically affect their ability to cultivate and maintain equitable and inclusive community partnerships. When segments of a community are discouraged from participating in exercise science research due to mistrust, a history of exclusionary recruitment practices, and ongoing fears regarding stigma or safety, an organization's ability to foster innovation is undermined.

Despite the growing body of health and medical literature describing TGD individuals' experiences with research participation (2, 6–9), no studies to date have explored TGD individuals' perceptions of, or comfort with participating in, exercise science research. As described above, many exercise science studies present unique contextual factors that may pose distinct participation barriers compared to other fields of health research. Without understanding how TGD individuals perceive these contexts, sport and exercise researchers risk perpetuating exclusionary practices and generating evidence that does not reflect the full diversity of a society. Therefore, the present qualitative study sought to explore factors influencing TGD individuals' willingness to participate in exercise science and the contexts in which they make participation decisions.

This study sought to identify actionable strategies to promote safer, more inclusive research practices and institutionally trustworthy research environments in exercise science and other kinesiology subdisciplines. Methodology for a Consensual Qualitative Research (CQR) approach was utilized to investigate an overarching research question: What factors influence, as barriers or facilitators, TGD individuals' willingness to participate in exercise research?

## 2 Materials and methods

### 2.1 Design and qualitative approach

Given the absence of prior literature examining how TGD individuals may perceive exercise research environments and recruitment materials, a qualitative approach was best suited to elicit meaningful insight from participants' lived experiences. We employed a CQR approach for this study to capture the depth and complexity of TGD individuals' perceptions of exercise research participation. We selected CQR because it is well suited to exploratory investigations in areas with limited prior literature and emphasizes inductive, consensus-based analysis grounded in participants' narratives (23–25). CQR combines constructivist and postpositivist elements through iterative analysis, multiple analyst perspectives, external auditing, and participant member checking to develop contextually grounded representations of participant experiences (24, 25). This participant-centered approach aligned with the study's goal of understanding how TGD individuals navigate decisions to participate in exercise science research.

The gender minority stress model (GMSM) served as a sensitizing framework in the early planning stages of the current study. The GMSM, an extension of minority stress theory, conceptualizes how external and internal stressors shape the lived experiences of TGD individuals (5, 26, 27). The GMSM informed the development of interview questions and provided conceptual grounding for how TGD individuals might perceive exercise science research. Prior research has demonstrated that expectations of stigma and experiences of discrimination shape TGD individuals' willingness to participate in research, perceptions of institutional trust, and comfort with being visible in unfamiliar settings (5, 28). Based on this, our questions focused on TGD individuals' comfort with common exercise research features, opinions on recruitment processes, and perceptions of exercise science methods, settings, and researchers.

### 2.2 Study participants

#### 2.2.1 Research team

Our research team presents a variety of ages, races, ethnicities, gender identities, and sexual orientations. While CQR articles often divulge specific details on study-relevant demographics for research team members, we shall not given the sensitive nature of our topic. On our team, some of us are TGD individuals; most of us have close personal ties to TGD individuals; all of us are allies who strongly support the lives, rights, and safety of TGD individuals. We acknowledge that our experiences and perspectives influenced the study design and analytic process. Reflexive attention to researcher positionality, participant relationships, and analytic interpretation remained an ongoing consideration throughout data collection and analysis. To support reflexivity and reduce interpretive bias, we used bracketing, consensus-based analysis, and external auditing throughout the study.

JDW, JWN, and JLM served as the primary research team. DWD, JDF, JDT, LEE, and WJS served as external auditors. All team members had prior exposure to qualitative methodology; although, experience conducting qualitative research and familiarity with CQR specifically varied across the team. Prior to data collection and analysis, JDW provided training and ongoing guidance regarding CQR procedures and analytic processes.

#### 2.2.2 Recruitment and selection

The Institutional Review Board of Western Kentucky University approved this study, including a broad recruitment process. We developed a screening survey using inclusive demographics questions (29), which we used to assess eligibility based on inclusion criteria (18 or older, TGD, in the United States, with ability to communicate in English). Participants were not required to have previous experience participating in exercise science research. Given that underrepresentation may reflect both exclusion and self-selection out of research, capturing perceptions from individuals without prior participation was essential to understanding barriers to engagement. At the end of the screening survey, volunteers provided an email address to be used for scheduling interviews. We compiled a list of every college and university in the United States with undergraduate or graduate programs in exercise science or related fields. At those institutions, we identified student and/or faculty lesbian, gay, bisexual, transgender, queer, intersex, asexual/aromantic/agender, and two-spirit (LGBTQIA2S+) organizations with publicly available contact information, as well as community organizations in locations surrounding those institutions. We provided those LGBTQIA2S+ organizations with study information, a link to the online screening survey, a flyer, and a copy of our IRB approval via email, asking that they share the information with members of their organization. We distributed the screening survey October–December 2024, and it remained open for responses until January 2025. Unintentionally, a degree of snowball sampling occurred, as multiple participants reported learning of the screening survey through social media or word of mouth.

Based on the screening survey, 54 eligible people volunteered to participate. Volunteers were stratified across the nine geographic divisions of the United States (New England, Middle Atlantic, South Atlantic, East South Central, East North Central, West North Central, West South Central, Mountain, and Pacific) (30). Within each division, participants were randomly selected and contacted iteratively until an adequate sample and geographic representation were achieved. Recruitment continued until *a priori* sampling goals were reached.

We set our desired sample size based on the CQR recommendation that samples be 8–15 participants (25). Additionally, we sought to achieve data saturation (the point during collection at which responses become redundant) and inductive thematic saturation (the point during analysis at which new analytic constructs no longer develop) (31, 32). *A priori*, we set our data saturation goal at two interviews in a row without new information from participants' responses; however, if that occurred before 15 participants, we would continue. For inductive thematic saturation, we assessed this retrospectively, determining at which point in analysis all domains and categories had developed. Despite these goals, after 13 interviews, we had only two transgender women or transfeminine participants. Thus, we decided to increase our sample size and refocused our selection efforts to increase the representation of trans women and transfeminine individuals in our sample. In total, 17 people participated in this study, with three others scheduling interviews but not completing them. Key self-reported demographic information is reported in Table 1.

**Table 1:** Participant demographics.

Category	*n*
Geographic Division	
Pacific	1
Mountain	3
East North Central	3
West North Central	2
New England	2
Middle Atlantic	2
East South Central	2
West South Central	2
South Atlantic	0
Age	
25 or under	11
Over 25	6
Education	
High school diploma	2
Undergraduate student	6
Master's student	3
Doctoral student	3
Master's degree	2
PhD	1
Self-Selected Race/Ethnicity	
White	12
Latino	1
Ashkenazi Jewish	1
Pacific Islander and East Asian	1
Middle Eastern	1
Gender Identity	
Transgender woman	2
Transfeminine	1
Transgender man	2
Man (transgender)	1
Transexual man	1
Nonbinary	4
Nonbinary trans woman	1
Nonbinary transfeminine	1
Nonbinary transmasculine	1
Bigender	1
Gender fluid	1
Nonbinary and apagender	1

Total *N* = 17. Age range = 18–59. South Atlantic division had no participants. Participants who did not identify as current students reported highest degree completed.

### 2.3 Data collection

#### 2.3.1 Interview guide

The interview guide was developed iteratively by the research team using open-ended questions informed by the GMSM and the study purpose. The guide underwent internal auditing, external review by a nonbinary researcher unaffiliated with the project, and review by TGD community members (*n* = 4). Two pilot interviews were conducted prior to formal data collection, leading to minor revisions to improve question clarity and reduce participant confusion. Table 2 presents the final interview guide.

**Table 2:** Semi-structured interview guide.

Included Questions
What do you know about sport and exercise science research? Aside from this study, what experience(s), if any, do you have participating in sport and exercise science research? What insights, if any, can transgender and gender diverse people bring to sport and exercise science by participating in research? What study recruitment materials for sport and exercise science research have you seen? What things about recruitment materials would make you want to volunteer for a sport and exercise science research study? If you had the opportunity to volunteer for a research study in sport and exercise science: What reasons would you have for volunteering (or not volunteering)? What specific types of studies would you volunteer to participate (or not participate) in? If you were participating in a sport and exercise science research study: What things about yourself, if any, would you be most willing to share with the researcher(s)? What things about yourself, if any, would you not be willing to share with researcher(s)? If you were participating in a sport and exercise science research study: What things about the study, if any, would make you feel uncomfortable, unsafe, or want to stop participating? What things about the study, if any, would make you feel comfortable, safe, or want to continue participating? If you were participating in a sport and exercise science research study: What things about the researcher(s), if any, would make you feel uncomfortable, unsafe, or want to stop participating? What things about the researcher(s), if any, would make you feel comfortable, safe, or want to continue participating? What are your opinions on transgender and gender diverse people participating in sport and exercise science research? What are your opinions on transgender and gender diverse people's safety while participating in sport and exercise science research?

#### 2.3.2 Interview procedures

Between January and May 2025, JDW conducted all interviews in a private, secure office using a university provided, FERPA and HIPAA-compliant, Zoom platform (Zoom Communications, Inc, San Jose, CA). At the start of each interview, JDW reviewed the study purpose, procedures, confidentiality protections, and participant expectations before obtaining verbal informed consent. Interviews followed a semi-structured, conversational format and ranged from 43 min to 1 h 47 min (median = 1 h 17 min) based on the depth of participants' responses. Participants received a $50 gift card following participation.

After each interview, JDW maintained a reflexive journal detailing brief interview summaries, perceptions of each interview, evolving perceptions of the data, topics and questions that arose in interviews that had not come from the interview guide, and introspection on the research topic and processes. This information was then shared with DWD to obtain additional comment and insight as a means of peer debriefing. Based on this reflexive and peer debriefing activity, by approximately the sixth interview, participants' narratives began to show convergence in major concepts and experiences, suggesting early informational abundance related to the primary research question. However, recruitment continued to support the study's *a priori* sampling goal of 15 interviews in order to ensure stability and depth of the data across cases. Additionally, after the 13th interview, targeted recruitment efforts were used to increase representation of transgender women and transfeminine participants within the sample.

Interview recordings were initially saved to the Zoom platform's cloud storage. Upon completion of an interview, JDW immediately destroyed video recordings. Recordings and transcripts were stored on secure, university-provided cloud storage (Google Workspace, Alphabet Inc, Mountain View, CA). JDW verified transcript accuracy, removed identifying information, and assigned pseudonyms prior to analysis. Participants were given the opportunity to review and edit transcripts before finalized transcripts were used for analysis. Fifteen participants confirmed reviewing their transcripts for accuracy, and four made changes or additions, which were incorporated into the final transcripts. We used these final transcripts for data analysis. Audio recordings and identifying materials were destroyed following transcript verification and member checking.

#### 2.3.3 Data analysis

CQR employs an iterative, consensus-driven analytic approached in which multiple analysts independently interpret participant narratives and then reach consensus regarding coding structure and data interpretation (25). Importantly, consensus in CQR refers to agreement among analysts rather than uniformity of participant perspectives. CQR analysis proceeded through stages involving domain development, domain coding, construction of core ideas, and cross-analysis to identify categories and subcategories across cases. Domains represented broad topic areas, core ideas reflected concise summaries of participant meaning within domains, and categories/subcategories captured shared patterns across participants. Internal and external auditing procedures were incorporated throughout the analysis to support rigor and trustworthiness. Analysis results are typically reported as a hierarchical structure of domains, categories within domains, and subcategories (where they exist) within categories. Additionally, labels are provided to describe how frequently each category or subcategory appeared across participants, and exemplar core ideas or quotes are selected to illustrate categories or subcategories.

We started data analysis in April 2025 after completing the ninth interview. JDW, JWN, and JLM served as analysts. JDF and LEE served as external auditors, reviewing the analysis at various points throughout the process. X4X, X6X, and X8X reviewed the results as external auditors after the complete analysis.

To identify domains, JDW, JWN, and JLM met and worked simultaneously through versions of the transcripts with the interviewer's questions and statements bracketed out. This allowed us to focus on the participants' words and meanings as we read the first transcript in whole, then line-by-line, to identify overarching topics from their narrative. As we identified a domain, we recorded its origin and definition. After identifying the first domain, we would determine if information fit the existing domain(s) or if a new domain had been identified. We continued in this manner as we reviewed the rest of the first transcript and for each subsequent transcript. We completed the first six transcripts together. After collaboratively analyzing initial transcripts, analysts independently completed subsequent transcripts before reconvening to resolve discrepancies regarding domain structure and complete the initial domain list. External auditors then examined the wording of domain titles, how adequately domains fit participants' narratives, and if smaller domains could be combined or if larger domains needed to be separated so that domains were as broad as possible while remaining distinct. After incorporating auditor feedback to rename some domains and combine multiple related domains, we finalized our domain list.

Using the domain list, we returned to the transcripts, with the interview questions no longer bracketed out, and blocked data into domains. This involved reorganizing chunks of information (i.e., blocks) from participants' responses into the most reflective domain. We domain blocked the first two transcripts together. From there, we used a rotating process for the remaining 15. Each analyst independently domain blocked 10 out of 15 transcripts; so that each transcript was independently domain blocked by two analysts. Then, the third analyst performed an internal audit of both versions of the five transcripts they did not domain block. This was undertaken to ensure participants' views were represented within the most appropriate domain and to address any disagreements between the two analysts who independently domain blocked the transcript.

With domains blocked, we constructed core ideas by abstracting and paraphrasing participants' responses as succinctly as possible while maintaining the original meaning within the context of the interview question. All three analysts developed core ideas for two transcripts together. Then, analysts worked in pairs to develop core ideas for the next nine transcripts, each pair analyzing three transcripts. Next, for the remaining six transcripts, analysts developed core ideas independently for two transcripts each. Finally, analysts internally audited the core ideas of any transcript for which they did not develop core ideas.

After developing core ideas within domains for each case, we began a cross-analysis. For each domain, we organized all core ideas across all cases into a single document. Working one domain at a time, we read through core ideas within a domain, identifying categories based on inductively derived patterns in core ideas across cases. When large amounts of data clustered within a single category, we repeated this process to identify variations and patterns across cases within the category that could be meaningfully clustered into subcategories. We continued this process for all domains. Next, we returned to the transcripts, reorganizing each case's domain blocked data and core ideas into the identified categories and subcategories, creating final “consensus-based” transcripts based on our analysis, which we returned to participants for further review. Using file access tracking features in Google Workspace, we confirmed that all participants opened their consensus-based transcript; however, only two provided comments, which primarily focused on requesting that specific quotes not be included in any published materials.

Finally, we applied frequency descriptions to subcategories based on the number of cases in which they appeared: general (appearing in 15 or more cases), typical (appearing in 9–14 cases), variant (appearing in 3–8 cases), or rare (appearing in 1 or 2 cases). When a category included no subcategories, we applied the frequency description to the category instead. These frequency classifications informed the framing and interpretation of findings by distinguishing broadly shared participant experiences from more individualized or context-dependent perspectives. Rather than repeatedly reporting frequency labels throughout the narrative results, we used them to guide the strength and breadth of interpretive claims within domains, categories, and subcategories. Exploratory subgroup analyses were conducted consistent with CQR recommendations; however, these analyses did not identify substantial differences in category frequency patterns across examined participant characteristics. Therefore, we do not report the results of these subanalyses as they did not add meaningful analytic information or context to the study's findings.

After the external audit, we returned final analytic results to participants for member checking. Although all participants accessed the results, none requested changes. Supporting inductive thematic saturation, all domains were identified from the first participant's transcript, all categories by the first two transcripts, and all subcategories by the fourth transcript. Suggesting stability of our findings across cases, most categories or subcategories had frequency descriptions of “general” or “typical.”

## 3 Results

From participants' narratives, we inductively derived five domains, 24 categories, and 43 subcategories. However, much of the data were unrelated to the primary research question. Given the breadth of participants' narratives, the presented findings focus on factors that shape willingness to participate in exercise science research and the research contexts in which those decisions are made. We made this decision in order to best represent those participant responses that directly answered the research question and purpose. Through our analysis, participants' narratives regarding factors that influence exercise science participation converged across three domains (Table 3): *Research Participation*, *Researcher Decisions and Behaviors*, and *Safety*. We present the hierarchical structure of these domains, the contained categories and subcategories, and the frequency characterizations of subcategories in Table 4. At least one exemplar quote illustrating relevant participant responses follows each described subcategory (or category).

**Table 3:** Reported domains.

Domain	Description	Selected quote(s)
Research participation	How TGD individuals describe their perspectives and beliefs that shape their internal decision-making regarding willingness to participate in exercise science research.	COLD PURPLE. So, volunteering to participate, you may want, on the one hand, to find out more about who you are as a person, and how all of humanity is because that's interesting. A lot of people have an instinct to want to answer these questions and see themselves represented in the research being done. But at the same time, you have to do that risk benefit analysis of if I am identified, what am I risking in order to have the benefit of being represented in the research?
Researcher decisions and behaviors	How TGD individuals describe actions, communication, and study design choices made by researchers that shape participants’ experiences and perceptions of inclusion, respect, and accessibility regarding exercise science research.	CELADON GREEN. One of the things that would make me more comfortable in exercise science research would be things like them being clear and forthright about what they want, what we're doing, all of that. Being respectful both to me and also of my time and effort. Being clear in communicating. I think an environment of mutual respect really helps research with human subjects.
Safety	How TGD individuals describe their broader sense of safety, vulnerability, and risk exposure as it relates to exercise science research participation and other life contexts.	FALCON VIOLET. I don't want to be the person that makes the news. I don't want to be the person that's scapegoated on Fox News after someone took a video of me participating in a study next to some cis woman like, look at this. That's not good. It's happened to girls though. And as a result of that, I'm kind of fearful of using public facilities. There's a lot of societal issues there. And so a gym is not exactly the safest place for me to be.

**Table 4:** Hierarchical structure of domains, categories, and subcategories.

Category	Subcategory	Characterization^a^
Domain: research participation		
Design components	Exercise Modalities Encouraging Participation	Typical
	Procedural Elements Encouraging Participation	Variant
	Exercise Modalities Encouraging Avoidance	Typical
	Procedural Elements Encouraging Avoidance	Typical
	Communication Elements	Typical
	Alignment with Identity	Typical
Personal comfort	Self-Perception	Variant
	Perceived Safety and Comfort	Typical
Perceived research context	Contextual Trust Appraisal	Typical
	Perceived Research Intent	Variant
Perceived benefits	Intrinsic Benefits	Variant
	Extrinsic Benefits	Typical
	Collective Benefits	Typical
Framing participation willingness		General
Domain: researcher decisions and behaviors		
Research design, data, and methodological considerations	Inclusive Participant Classification	Typical
	Respectful Data Collection Procedures	Typical
	Data Privacy and Security	Variant
	Participant Safety and Comfort Accommodations	Typical
Inclusive recruitment strategies	Recruitment Materials	Typical
	Recruitment Channels and Messaging	Typical
	Recruitment and Sampling Strategies	Typical
Researcher identity, competence, and conduct	Researcher Knowledge, Training, and Preparedness	Variant
	Research Team Identity and Representation	Typical
	Interpersonal Conduct and Communication	General
Domain: safety		
Sociopolitical climate risks	Geopolitical Threat Perception	Typical
	Sociocultural Stigma and Hostility	Typical
Safety appraisal of researchers and institutions		General
Privacy and disclosure risks		General
Setting and procedural safety		Typical
Lived safety appraisal		Typical

a Characterization based on the frequency with which the subcategory or category appeared across participants:
General = appeared in 15 or more cases Typical = appeared in 9–14 cases Variant = appeared in 3–8 cases

### 3.1 Domain 1: research participation

This domain captures how TGD individuals describe the internal decision-making processes that shape their willingness to volunteer for and participate in exercise science research. This domain and its categories reflect how participants interpret, evaluate, and respond to research elements based on their lived experiences and perceptions. This domain includes the categories *Design Components*, *Personal Comfort*, *Perceived Research Context*, *Perceived Benefits*, and *Framing Participation Willingness*. Across categories, participants described weighting study design features, contextual factors, and personal comfort to decide whether to participate. This domain illustrates that willingness to participate is constructed through complex, ongoing appraisal of research opportunities.

#### 3.1.1 Category 1.1 design components

This category reflects the ways in which a study's structural and methodological features influence TGD participants' willingness to take part. Participants described how decisions to participate are shaped by the types of exercise included (subcategories: *Exercise Modalities Encouraging Participation/Avoidance*), the procedures required to complete the study (*Procedural Elements Encouraging Participation/Avoidance*), how information about the study is communicated (*Communication Elements*), and whether the study design and eligibility criteria affirms TGD identities (*Alignment with Identity*). Across subcategories, participants described evaluating design components in relation to their interests, comfort, and sense of inclusion, with willingness to participate increasing when studies felt relevant and affirming.

##### 3.1.1.1 Subcategory 1.1.1—exercise modalities encouraging participation

Participants revealed specific exercises and activities that would facilitate exercise science participation. Specific activities varied widely across individuals; however, the overarching theme of the subcategory was that TGD individuals would volunteer to participate in exercise science studies involving exercises, activities, and/or sports they already engaged in or enjoyed.

CELADON GREEN. I would be interested in participating in studies when they're about things that I potentially find interesting or involved doing things that I appreciate doing. For instance, lately I've been biking more… Weightlifting would also be in that category. I lift so that definitely helps. Studies on exercise activities which I have experience with and enjoy doing definitely helps.

##### 3.1.1.2 Subcategory 1.1.2—procedural elements encouraging participation

Several participants described being more willing to participate in exercise science studies when procedural elements reduced uncertainty, burden, and perceived risk. For some participants, convenience, accessible research settings, and alignment with participants' schedules shaped decisions about participation. These participants described a preference for studies that minimized logistical demands.

DEEP SKY BLUE. My willingness to participate depends on the nature of the study, and the convenience of participating. Probably the less commitment the more likely I'd be able to do it. It's just very practical things about scheduling amidst other obligations.

##### 3.1.1.3 Subcategory 1.1.3—exercise modalities encouraging avoidance

Similar to those activities that would facilitate participation, most participants described uninteresting, unenjoyable, or overly strenuous activities as barriers to participation. Participants most often cited running as a specific example. Additionally, one participant noted that general exercise behaviors influence participation willingness.

FIREBRICK RED. There are a lot of trans people who don't feel comfortable going to the gym, and that is a significant barrier to exercise. I think barriers to exercise definitely carry over as barriers to participating in exercise research.

##### 3.1.1.4 Subcategory 1.1.4—procedural elements encouraging avoidance

Participants identified a variety of methods viewed as invasive, discomforting, or exclusionary as barriers to research participation. Most frequently, participants identified any data collection that involved piercing the skin (e.g., blood draws, muscle biopsies) as a reason they would not participate in an exercise science study. Contrasting elements encouraging participation, participants described inconvenient or overly time-consuming study procedures as decreasing willingness to participate.

GOTHAM GREEN. You're taking time out of your day to basically be poked and prodded at for being a trans person, and that's exhausting. Even if it's done with the best intentions, it's exhausting.

##### 3.1.1.5 Subcategory 1.1.5—communication elements

Participants described how communication, particularly within recruitment materials, shaped decisions about participation. Participants were more willing to engage with exercise science studies when messaging used transparent, inclusive, and straightforward language that allowed them to quickly determine eligibility, purpose, and expectations without additional effort. Clear and explicit communication reduced uncertainty and helped participants assess whether participation felt accessible, worthwhile, and appropriate to pursue. In contrast, vague wording, ambiguous eligibility, and messages that required participants to infer whether TGD individuals were welcome contributed to frustration, hesitation, and disengagement with research opportunities.

FIREBRICK RED. I think I would participate in a lot more studies if they were clear with what they meant by sex. I saw a flyer for a study on male pattern baldness that I could have benefitted from given my male hormone profile, but I couldn't tell from the eligibility criteria if I would be allowed because they weren't explicit with what exactly they meant. That prevented me from seeking it out further.

##### 3.1.1.6 Subcategory 1.1.6—alignment with identity

Willingness to participate increased when study materials, eligibility criteria, and procedures clearly communicate that TGD individuals are intentionally included, accurately categorized, and genuinely valued as part of the target population. Participants described feeling discouraged, despite interest in the research topic, when recruitment language relied on sex-based criteria, centered cisgender participants, or created uncertainty about where TGD participants “belonged” in the sample.

ELECTRIC CRIMSON. I wouldn't want to participate if there are pretty strict sex categories in terms of participation. I think that if there were something where it's like, “Oh, you're categorized as female [instead of as a man].” I don't think that would fully represent my body or my situation at all. So, I think that would kind of be where I would have questions. But I think that obviously, if there's that categorization, usually the study's excluding trans people to begin with.

#### 3.1.2 Category 1.2 personal comfort

This category captures how participants’ internal sense of physical, psychosocial, and identity-related comfort shapes their decisions whether to participate in exercise science research. The contained subcategories reflect two interrelated dimensions of this construct: *Self-Perception*, centered on participants’ internal experiences of their bodies, and *Perceived Safety and Comfort*, reflecting how participants appraise potential risks within research.

##### 3.1.2.1 Subcategory 1.2.1—self-perception

Several participants described willingness to participate in exercise science research as influenced by internal bodily comfort, gender affirmation, and self-efficacy. While several participants discussed their regular exercise behaviors, some participants described self-excluding from exercise science research due to internal judgments related to fitness or physical activity levels.

SCOOTER BLUE. I do believe, and I'm speaking from personal experience, that there might be this expectation from trans people who are less active of not being able to contribute to an exercise science research study because they're not active. I think from that, there might be barriers there where it's almost a feeling of inadequacy that keeps them from participating.

Dysphoria, body image concerns, transition status, and access to gender-affirming care shape comfort with exercise and sharing personal information. Participants noted that feeling “out of shape” or uncomfortable in their bodies could reduce willingness to participate.

BRIGHT FUCHSIA. The idea of being watched or monitored while I'm working out already makes me not want to participate. I already feel vulnerable working out because I'm not as comfortable in my body as I would like to be. If I'm being watched, I think then I'll be more uncomfortable. I do work out, doing things I enjoy like going for walks outside, but I think I would feel that I was being judged if I were doing it in a more clinical setting.

Participants often acknowledged that similar body image and exercise-related concerns were not unique to TGD individuals but may feel compounded by the context of marginalization.

COLD PURPLE. I'm not even sure that's all that different from cis men and cis women who also have very similar complicated issues with their body and their self and their mental image of themselves.

##### 3.1.2.2 Subcategory 1.2.2—perceived safety and comfort

Participants described that perceived risk of harm, fear of data disclosure, and comfort with physical exposure requirements for data collection may discourage TGD individuals from participating. However, participants also described that research that felt accommodating, respectful, and nonexploitative on these topics would likely encourage participation despite these initial concerns.

GOTHAM GREEN. I have found that biology, broadly, and physiology, specifically, kind of still has a lot of transphobia that's built into it. I always get nervous about participating in those kinds of studies because I am afraid that I will run into someone who has very specific negative ideas of what it means to be a trans person. I'm just worried that I'd run into someone transphobic, and it would suck.

#### 3.1.3 Category 1.3 perceived research context

This category captures how participants evaluate the broader ethical and sociopolitical context surrounding a research study, including the setting and those conducting the research. Participants described their evaluation processes in two key dimensions: *Contextual Trust Appraisal* and *Perceived Research Intent*. Participants described how they synthesized their perceptions of researchers, institutions, and communities to determine if research feels legitimate, respectful, and safe. Participants also interpreted the underlying purpose and motivations of a study.

##### 3.1.3.1 Subcategory 1.3.1—contextual trust appraisal

Participants described evaluating the trustworthiness and perceived safety of research opportunities through a synthesis of perceptions regarding researchers, institutions, sociopolitical conditions, and community validation. Perceptions of allyship, respect, and transparency interacted with broader contextual factors to shape participation willingness. Community networks and shared experiences served as signals of legitimacy and safety, which further reinforced or undermined trust in an exercise science research opportunity.

WATTLE GREEN. If I saw that the study came from an organization that had done discriminatory things in the past or had recently had something that negatively affected trans people, I would be hesitant to participate.

##### 3.1.3.2 Subcategory 1.3.2—perceived research intent

For several participants, perceived purpose and intent of a study influenced willingness to participate. Participants were more willing to participate when study goals were clear, meaningful, and novel, but hesitant when goals seemed vague, industry-driven, or redundant. Some participants evaluated participation decisions in the context of whether they believed the research would benefit or potentially harm the TGD community.

CHESTNUT ROSE. I wouldn't participate if I got the sense of someone collecting data in hopes to change or exploit gender diverse individuals or the needs of those individuals. I think if it seemed like the research was happening in a way, and questions were being asked, that didn't seem like it related to or benefitted gender diverse individuals in some aspect, I would stop participating.

#### 3.1.4 Category 1.4 perceived benefits

This category reveals ways participants evaluate the positive outcomes associated with participating in exercise science research and reflects how perceived benefits increase willingness to participate. Participants described these benefits across three complementary forms of motivation: *Intrinsic Benefits* rooted in intangible reward of the experience, *Extrinsic Benefits* tied to tangible rewards, and *Collective Benefits* focused on contributing to the TGD community.

##### 3.1.4.1 Subcategory 1.4.1—intrinsic benefits

Several participants described curiosity, personal interest, and a desire to learn as intrinsic motivations for participation in exercise science research. Many participants viewed research participation as enjoyable, meaningful experiences that could provide insight into their health or physical abilities.

LAVENDER ROSE. I'm fairly certain that a lot of trans people do want or would want to participate in exercise science, either out of sheer curiosity or out of a genuine desire to have that knowledge brought back into existence for the sake of science. At least for me, that's a big one.

##### 3.1.4.2 Subcategory 1.4.2—extrinsic benefits

Whereas some participants emphasized curiosity and personal meaning, many participants described compensation and practical benefit as important. Multiple participants explained that monetary compensation was a meaningful motivator, particularly when it offset missed work or supported those with limited resources. Practical benefits such as useful health information, clear physical performance outcomes, and convenient opportunities for exercise also increased interest.

SHAMROCK GREEN. There's the noble reason of wanting to further the scientific field in exercise and wanting to do my part to hopefully help things be more inclusive or accommodating for people of the trans persuasion in the future. But then, also, getting paid is really nice too.

##### 3.1.4.3 Subcategory 1.4.3—collective benefits

Participants described their willingness to participate in exercise science as influenced by the perceived value of their contribution to the broader TGD community. Unlike the *Intrinsic Benefits* rooted in personal curiosity or individual enjoyment, or the *Extrinsic Benefits* rooted in rewards from outside the research experience, perspectives in this subcategory reflect an outward orientation toward advancing representation, improving health equity, and contributing to information that may benefit other TGD individuals. Participants emphasized that research was more meaningful when addressing knowledge gaps centered on TGD experiences and had the potential to inform more inclusive practices. Decisions to participate were often contingent on a study being perceived as contributing to the community rather than extracting data without clear benefit.

CHESTNUT ROSE. Elder trans role models are rare. But that role model representation has to come from somewhere, and I think a lot of gender diverse people who have done that reflection want that. So, they are willing to participate because they know that it's important, and they know the impact it could have.

#### 3.1.5 Category 1.5 framing participation willingness

Nearly all participants described willingness to participate in exercise science research as a dynamic, context-dependent process expressed across personal, conditional, and community-referenced frames. Participants articulated their willingness through a combination of individual readiness, situational requirements, and assumptions about collective TGD experiences. Many participants framed their own decisions in relation to perceived community patterns, contextualizing their willingness using shared identity and vulnerability.

DEEP SKY BLUE. Willingness to participate in exercise science research depends on the nature of the study and individual preferences. There are trans folks who are super sporty and some who are not, and it's just individual preference. Just as it would for a cisgender individual, people will vary on whether they'd want to participate, why they would want to, and what they'd be willing to do.

### 3.2 Domain 2: researcher decisions and behaviors

This domain captures how participants describe researcher-controlled actions that shape TGD participants experiences during and after enrollment in exercise science research, oriented toward external factors guided by researcher decisions. Participants frequently framed their narratives in terms of practices that (1) they wished to see adopted by researchers, (2) would lead them to discontinue participation, or (3) they believed would increase willingness to volunteer and/or continue participation. This domain includes the categories *Research Design Considerations*, *Inclusive Recruitment Strategies*, and *Researcher Identity, Competence, and Conduct*. Participants evaluated these elements as observable signals that researchers are prepared to engage with TGD individuals respectfully and responsibly. This domain reflects researcher-controlled actions as ongoing modifiable factors that shape participant experiences, distinct from the internal appraisals that guide initial willingness to participate.

#### 3.2.1 Category 2.1 research design considerations

This category reflects participants' perspectives on how researcher decisions regarding study design and implementation shape participation experiences. Participants focused on procedural and methodological choices by researchers that influence TGD participants' safety, comfort, and affirmation while participating. Here, participant narratives converged across four subcategories: *Inclusive Participant Classification*, *Respectful Data Collection Procedures*, *Data Privacy and Security*, and *Participant Safety and Comfort Accommodations*. Across these subcategories, participants underscored that design decisions actively signal whether an exercise science study is prepared to engage TGD individuals as participants.

##### 3.2.1.1 Subcategory 2.1.1—inclusive participant classification

Participants stressed that demographic and identity data must avoid binary assumptions and misclassification, particularly grouping by sex assigned at birth. Participants viewed inclusive gender options, pronoun use, transparency in analytic decisions, and sensitive collection of transition and gender-affirming care data as essential.

FIREBRICK RED. I know that I've taken part in some research surveys where they will ask about sex assigned at birth, and then they will ask about gender identity separately. You know it's a good effort to be cognizant of these things, but then they will make the gender identity categories mutually exclusive, like, you're either man, woman, or transgender, or maybe nonbinary. That definitely is just bad vibes because it's like, okay, if I answer transgender, are you saying I'm not a man? Are you saying I can't be both? It feels like at that point I have to misgender myself to participate.

Beyond inclusive demographic options, participants often discussed how best to incorporate TGD individuals into commonly used, binary sex-based statistical analyses. Most participants expressed that TGD should be included in sex-based analyses based on their gender. Additionally, some participants recommended allowing nonbinary participants (or all TGD participants) to self-select their preferred group for any sex-based analyses.

FIREBRICK RED. In a sex-based analysis, I think just including trans people in the binary sex categories based on their gender instead would be ideal. I know that there can be some concerns if your study really is trying to examine different hormonal profiles and kind of using sex as a proxy for that. Depending on the stage and transition that someone's at, they might not fit into that profile that you're looking for. But I think that a lot of the time—even most of the time—it should be okay to just put trans men with the other men, trans women with the other women. If you need to, run some sensitivity analyses after that, then.

However, other participants suggested doing away with sex-based analysis in favor of other group classification criteria, such as hormone levels or anthropometric measures.

RED VIOLET. My very first thought is just that it's interesting that we do testing based off of males vs. females in the first place because that kind of ignores a lot of aspects… I feel my first instinct is just throw aside the idea of doing things based off of male and female, and instead, use something like BMI. Well, maybe not BMI. Do it based off something different. Make it more specific like muscle mass percentage or a height-to-weight-to-athleticism ratio or whatever. Do it in different ways. If you want to test something like male vs. female, there's probably some blood test or some sort of genetic testing that could be used instead.

##### 3.2.1.2 Subcategory 2.1.2—respectful data collection procedures

Participants emphasized that data collection should be necessary, justified, and sensitively framed. Participants stressed that researchers should be transparent about what, how, and why data are collected, and that collection should protect privacy, minimize exposure, and safeguard autonomy. Additionally, participants recommended researchers avoid invasive or irrelevant data collection, especially that which could cause stigma or dysphoria.

DEEP SKY BLUE. That's really the main thing; being very careful about not objectifying people. But again, these are things you need to do all the time with all participants.

GOTHAM GREEN. Don't collect unnecessary information. Like not collecting unnecessary identifying data. I'm sorry, but I'm not giving you my phone number if it's clear to me that the study doesn't actually need it. Things like that.

Overall, participants described designing research with TGD participants in mind rather than as an afterthought as essential to participant dignity and safety.

##### 3.2.1.3 Subcategory 2.1.3—data privacy and security

Several participants explained that strong confidentiality protections are essential due to the risks of reidentification and data misuse for TGD individuals. These participants often emphasized legal compliance, data deidentification, data security, and transparency about data use and dissemination. For these participants, ethical intent and preventing data from being weaponized against TGD communities were also viewed as critical safeguards.

GOTHAM GREEN. When trans people are a target demographic, and it's clear the government has no issue accessing information they should not have, I just think that now, more than ever, trans people's data needs to be deidentified as much as possible during research. Otherwise, there's the inherent lack of safety in being out to a large group of people.

##### 3.2.1.4 Subcategory 2.1.4—participant safety and comfort accommodations

Participants described the need for trauma-informed, privacy-protecting study procedures that reduce dysphoria and preserve autonomy, including flexible data collection options, private spaces, and ongoing safety check-ins. Participants emphasized sensitivity to transition status and gender-affirming devices (e.g., gaffs or binders), as well as proactive management of external risks. Further, many participants described involving TGD individuals in a study's design as essential to respectful, safe research.

ELECTRIC CRIMSON. I think researchers should be flexible in terms of options. Like if there's specific clothing people have to wear when participating, have multiple options. If someone needs to wear a heart rate monitor, maybe give them a tutorial on how to do it themselves. Just really having options for what participants feel okay with. I think a lot of trans people would be willing to participate if they were asked what accommodations they would like to have to make sure that they feel comfortable. I think just being asked could be so valuable.

#### 3.2.2 Category 2.2 inclusive recruitment strategies

This category reflects participants' views on how researcher-driven recruitment decisions shape whether TGD individuals are exposed to research opportunities and feel safe considering participation in exercise science research. This category focuses on how recruitment design and execution impact willingness rather than interpretation of recruitment materials during individual decision-making. Participants described recruitment as a multi-component process involving the development and message of materials (*Recruitment Materials*), selection of modes and dynamics of dissemination (*Recruitment Channels and Messaging*), and strategic approaches to identify and safely engage participants (*Recruitment and Sampling Strategies*). Participants framed recruitment as an active researcher responsibility, where message and messaging decisions determine whether TGD individuals perceive research opportunities as legitimate, accessible, and safe.

##### 3.2.2.1 Subcategory 2.2.1—recruitment materials

Participants believed recruitment materials should be clear, professional, and visually engaging. Content of recruitment materials should be written in plain language with explicit statements about purpose, eligibility, time commitment, benefits, and confidentiality. Participants considered inclusive language and visible representation of TGD individuals as essential. Participants felt materials that signaled safety, transparency, and genuine benefit to the TGD community were the most likely to promote trust and participation.

COLD PURPLE. For this context, in your recruitment, make it clear that you're interested in men, women, trans, nonbinary, gender diverse; you're interested. Make it clear that when you say that you want anybody 18 to whatever the age bracket is and whatever the other eligibility criteria are, that you mean, “Yes, you too. We're not excluding you.” We want you to make sure that when we read your criteria, we feel like we do fit in that. If I feel that I fit in the criteria, both denotationally and connotationally, and my participation is clearly desired, I'm fairly likely to participate. But I need to be sure that I'm the target demographic, not adjacent to that.

##### 3.2.2.2 Subcategory 2.2.2—recruitment channels and messaging

Participants described recruitment channel effectiveness as dependent on age, platform, and safety. Social media was often effective for younger participants, though some preferred email or entirely avoided online spaces. Participants viewed flyers in LGBTQIA2S + affirming spaces or community locations as safe and useful. Overall, participants emphasized intentional, TGD-specific outreach that prioritizes safety as the most effective approach to distribute recruitment materials.

RED VIOLET. In-groups would be very effective… Historically, I always felt better about things when I would hear about it from a person that I knew as opposed to seeing a post online. If somebody is telling you, “Hey, I am participating in this thing…” then I feel more like there are real people behind it. I feel more incentivized to participate, especially if I hear from people who are like me, who have similar experiences to me.

##### 3.2.2.3 Subcategory 2.2.3—recruitment and sampling strategies

Participants described recruitment as effective when mediated by trusted LGBTQIA2S+ organizations, community networks, and snowball sampling. Participants expressed that recruitment and sampling should emphasize inclusivity and representation to avoid narrow or exclusionary targeting. Confidentiality and protection from harm were central concerns for most participants.

FIREBRICK RED. I think collaborating with queer and trans organizations is a good idea. I think kind of making it almost like community based participatory research in some way, allowing that community input.

#### 3.2.3 Category 2.3 researcher identity, competence, and conduct

This category describes how participants evaluate researchers as determinants of their perspectives on participation. Participants described evaluating researchers across three interrelated subcategories: *Researcher Knowledge, Training, and Preparedness*; *Research Team Identity and Representation*; and researchers' *Interpersonal Conduct and Communication*. Participants stressed that willingness to participate is shaped by what researchers do, who participants perceive them to be, and how they engage with participants. These factors informed judgments about credibility, cultural competence, and trustworthiness.

##### 3.2.3.1 Subcategory 2.3.1—researcher knowledge, training, and preparedness

Several participants expressed that researchers must demonstrate cultural competence, apply inclusive language, and understand the diversity of TGD experiences. Participants reported that researchers working with TGD participants should first complete training on sensitivity towards TGD individuals, cultural competency related to the TGD community, and trauma-informed research practices. Participants viewed trauma-informed practice, transparency, awareness of participant discomfort, and avoidance of misinformation as essential to building trust. Interdisciplinary teams were seen as better prepared to conduct affirming research with TGD participants.

BRIGHT FUSCHIA. I think there's definitely considerations that need to be made. Having folks that are culturally competent as researchers and have done training with gender diversity and know how to refer to people in ways that are not going to give them dysphoria, I think, is extremely critical, and a huge part of safety, even beyond physical safety.

##### 3.2.3.2 Subcategory 2.3.2—research team identity and representation

Participants reported greater comfort when research teams include LGBTQIA2S+ members and reflect diversity of identity, body type, and lived experience. Homogeneous teams were viewed as less welcoming, while visible allyship and affirming self-presentation signaled safety.

FALCON VIOLET. I think having trans people on the research team is an excellent way of ensuring greater participation. Knowledge that part of our community is represented on the team that's doing the research, is involved in the research in some way, really is an excellent way of ensuring that we have a greater sense of trust in that. If one of our community is already signed off on it, we're much more likely to be willing to be part of it. It puts a lot of us at ease to know that someone we can connect with personally is on the team.

##### 3.2.3.3 Subcategory 2.3.3—interpersonal conduct and communication

All participants felt that respectful, transparent, and affirming communication is essential for comfort during participation. They valued clear explanations of collection procedures and data use, ongoing updates, inclusive language, professionalism, and attempts to develop genuine rapport. Disrespect (especially misgendering participants) and vague or overly clinical language eroded trust and could lead to study withdrawal.

ANZAC ORANGE. If the researchers routinely misgender me or other participants is probably the biggest thing that would make me uncomfortable. Or if they just ask insensitive questions like, “So, what's in your pants?” Or the “Oh, my gender is a helicopter,” type of bad jokes. Things like that. I think misgendering is the biggest one though.

### 3.3 Domain 3: safety

This domain and its categories describe how participants conceptualize safety, vulnerability, and risk as part of their broader lived experience as TGD individuals. Rather than reflecting discrete decisions about participation willingness, this domain captures how underlying perceptions shape safety interpretations across contexts and environments. Participants described interaction among multiple factors establishing a baseline sense of risk that extends beyond any single research opportunity. This broader sense of risk established an analytical framework through which participants appraised research opportunities to inform decisions about participation.

#### 3.3.1 Category 3.1 sociopolitical climate risks

This category reflects how participants describe broader sociopolitical conditions that shape their sense of safety and vulnerability. Participants identified two subcategories: structural threats arising from laws and political jurisdictions (*Geopolitical Threat Perception*) and social threats arising from stigma and public discourse (*Sociocultural Stigma and Hostility*). Participants described these forces as interacting layers of risk that extend beyond individual research settings, shaping baseline perceptions of safety that ultimately influence comfort with participation in exercise science research.

##### 3.3.1.1 Subcategory 3.1.1—geopolitical threat perception

Participants described TGD general safety as strongly location-dependent and shaped by political climate. Many stated governmental policies directly affected their willingness to disclose their identity or participate in research, particularly in locations with fewer protections. Participants viewed geopolitical context as a structural force influencing both perceived and actual safety.

LAVENDER ROSE. Legality is actually a concern. Legality of identity is a big one. Access to hormone therapy is another big one, especially legality of access to hormones. It's also about the legality of my presence within the state or city or country that I'm in. I wouldn't participate in a study in Tennessee because it would be illegal just to be myself in such an area.

##### 3.3.1.2 Subcategory 3.1.2—sociocultural stigma and hostility

Participants perceived the U.S. sociocultural climate as characterized by politicization, sensationalism, and hostility toward people in the TGD community, especially in sport and exercise settings. Many participants expressed fears of backlash, scapegoating, or harm, noting that being openly trans often feels unsafe. Participants viewed media narratives, scientific disinformation, transphobia, and transnegativism as reinforcing collective TGD vulnerability despite some perceptions of individual safety.

CELADON GREEN. Right now, being openly trans is a bit like having a target painted on your back. So I can see why people would be reluctant to reveal that kind of thing. I see why someone would be hesitant to reveal that or participate in studies that could inadvertently reveal things.

#### 3.3.2 Category 3.2 safety appraisal of researchers and institutions

Nearly all participants described safety as contingent upon their appraisal of the people and institutions responsible for conducting research. Participants consistently evaluated researchers as representatives of broader institutional trustworthiness and safety. These appraisals were informed by perceived motives, institutional reputation, prior actions, and visible alignment with TGD safety needs. Participants described using these judgments to determine whether engagement with a study felt fundamentally safe, reflecting a higher-level evaluative process centered on trust in those conducting the research.

WATTLE GREEN. Honestly, if I saw recruitment for a study on trans people from the university where I go, I would be kind of hesitant, or I would at least want to look more into the researchers because our university has a complete DEI ban, no legal recognition of trans people, like we have a bathroom ban here. So if an organization like that was sponsoring the study, I would be a little bit questioning their motives.

#### 3.3.3 Category 3.3 privacy and disclosure risks

All participants expressed decisions to disclose personal information as an ongoing, conditional risk-benefit appraisal shaped by perceived relevance, privacy protections, and potential consequences of identification. Concerns regarding privacy, involuntary disclosure, and visibility risks emerged across nearly all participants. While most participants expressed willingness to share information commonly collected in exercise science research, they described hesitation to disclose data perceived as unnecessary, highly sensitive, or potentially identifying. Concerns about reidentification, data misuse, and the small size of the TGD population heightened hesitancy.

GOTHAM GREEN. I don't want to give researchers all this personal information about me. In the current political climate, it is dangerous to be trans. Then, combine that with participating in a study where all of my personal information can be included together with the fact that I'm trans. There's no tangible benefit for me in that.

#### 3.3.4 Category 3.4 setting and procedural safety

Participants explained that safety during participation is contingent upon the immediate physical and procedural context of the research. Narratives emphasized how characteristics of the setting, such as spatial layout and ease of egress, shaped perceptions of safety. Participants described appraising safety in real time based on how the environment and procedures interacted with identity-related vulnerability.

CHESTNUT ROSE. I also think about physical privacy and safety like when I said earlier that I have an exit strategy. If I have to go into this building, through this entrance, these three doors down this hallway, then up these stairs, etc. That doesn't lead to a comfortable space for me.

#### 3.3.5 Category 3.5 lived safety appraisal

Participants described navigating environments as a TGD person as an ongoing, internal evaluation shaped by past trauma, identity-related vulnerability (such as dysphoria), and awareness of broader community risk. Narratives reflected patterns of hypervigilance, self-monitoring, and anticipatory caution extending across everyday contexts, including research participation. Participants noted that they filtered their decisions to engage with exercise science research through these lived safety frameworks. Within these frameworks, factors such as identity and visibility carried both affirming and threatening implications. Participants framed their willingness to participate as shaped by the conditions of a given study within the contexts of these pre-existing, lived experience-driven perceptions of safety.

COLD PURPLE. I feel like there are certain elements that tighten it just a bit for non-cis folks, and part of that is because of our relationships with ourselves. But part of it is, I think, comfort at being seen by the public as ourselves because very often we're told that we shouldn't exist, or we don't exist. So, being seen for ourselves is simultaneously a really good thing—and it feels gratifying to be recognized—but also, there is an instinctive fear built up over years that the way to survive is to avoid being seen.

## 4 Discussion

Our findings indicate that TGD individuals’ willingness to participate in exercise science research is shaped by interconnected structural, interpersonal, and procedural factors. Participants described weighing intrinsic curiosity and potential benefits against concerns about transphobia, physical harm, confidentiality violations, and institutional distrust. Notably, many desired accommodations and protections consistent with best practices in human subjects research yet emphasized that such safeguards carry amplified importance for TGD participants. These findings align with prior research showing TGD participation in health and clinical research is contingent upon perceptions of trustworthiness, inclusion, and safety rather than solely on topic interest or potential compensation (8, 33, 34). Participants in the present study described community-mediated trust, visible allyship, and transparent communication as effective facilitators of participation in exercise science, similar to previous clinical research (6, 34). This study builds upon existing literature by situating these dynamics specifically within exercise science, a discipline where historically, research has been often structured around binary sex classifications and comparisons (2, 35).

This study advances exercise science and sport policy scholarship by demonstrating that participation in exercise science research is shaped by individual decision-making within a framework that considers institutional trust, perceived legitimacy, and sociopolitical safety. Findings suggest that exclusionary research structures and mistrust may contribute to inequities in sport-related knowledge generation by limiting the representativeness of evidence used to guide sport, physical rehabilitation, and public health practices. By extension, this constrains organization's ability to develop structured programs that optimize training and health benefits to diverse athletes and other service recipients. The findings of this study have implications for organizations, in terms of their prevailing norms, policies, and practices, which could either foster inclusion of diverse gender groups or stifle it.

### 4.1 Centrality of safety

Mistrust and distrust emerged as a pervasive theme across domains. Mistrust is an often passive, general feeling of unease or suspicion arising from anxiety or uncertainty, while distrust is an active lack of trust arising from past experience or specific knowledge of wrongdoing. Participants assessed research opportunities through an ongoing appraisal of physical, emotional, and social risk above all other considerations. Safety concerns extended beyond study procedures to encompass researcher demeanor, institutional affiliation, data misuse, and potential misinterpretation of study results. This aligns with previous research demonstrating that trustworthiness (rather than trust alone) drives participation of historically excluded groups in research (33). Participants described evaluating “vibes,” transparency, and professionalism when deciding whether to engage in exercise science studies, echoing findings that interpersonal cues shape perceived safety for those in marginalized communities (34). Participants' narratives suggest that perceptions of safety extended beyond physical or interpersonal comfort and reflected broader evaluations of institutional legitimacy, and the environments they fostered. These calculations were used to assess individual vulnerability within sport and exercise systems. Participants frequently interpreted recruitment materials, eligibility criteria, data handling, and researcher conduct as indicators of whether institutions, themselves, were prepared to engage TGD individuals ethically and safely. In this context, researcher decisions and behaviors functioned as signaling broader institutional practices and values.

Minority stress frameworks suggest that chronic exposure to discrimination fosters anticipatory vigilance and hyperawareness of institutional risk (26). Although we did not analyze data deductively through the GMSM or minority stress theory, participants' narratives reflect distal (structural), interpersonal, and proximal (internalized vigilance) stress processes influencing participation decisions that align with constructs from minority stress theory. Importantly, safety as described by our participants was not limited to protection from physical harm. Rather, participants described safety as multidimensional, encompassing bodily, psychosocial, political, and institutional safety alongside protection against epistemic harms such as misclassification, erasure, or misuse of participant data. To our participants, safety included psychological affirmation, avoidance of dysphoria, and protection against reidentification. Given the relatively small size of TGD populations, concerns about data security were heightened, which is consistent with literature documenting that TGD individuals experience privacy anxieties in research contexts (2).

Collectively, these findings highlight that safety is not a discrete variable but an interpretive framework through which TGD individuals evaluate research, and most things in life. Exercise science researchers must therefore understand safety as relational, conditional, and contextually constructed rather than as solely related to procedural compliance with ethical research regulations. Organizational leaders and administrators must understand and commit to trainings and infrastructure policies which enable researchers and project personnel to foster social, psychological and politically safe climates in tune with the concerns of TGD individuals.

Although participants demonstrated substantial convergence regarding the importance of safety, trust, respect, and inclusive research practices, variation emerged in how participants prioritized these factors when evaluating participation decisions. Some participants described extensive concerns regarding political visibility, misclassification, and institutional mistrust, whereas others framed participation decisions more pragmatically around convenience, logistical burden, or compensation. Other participants described a combination of these perspectives, viewing different aspects of their decision-making through multiple lenses. These differences suggest that while inclusive and affirming research practices may function as foundational expectations for many TGD individuals, participation decisions remain individualized and shaped by prior experiences, transition-related contexts, and perceived vulnerability within exercise and research environments.

### 4.2 Barriers to exercise science participation

Barriers described in the current study mirror those identified in broader health research. Participants cited invasive procedures, unclear purpose, logistical burden, and exclusionary eligibility criteria as barriers to participation. In clinical research, TGD individuals report similar hesitations regarding laboratory tests, misclassification, and protocol elements that fail to account for gender-affirming care (2). Exercise science adds unique considerations such as discomfort with gym or sports environments, fear of observation, and concerns about public bodily scrutiny. These factors have been documented as barriers to physical activity participation among TGD individuals (16), which several of our participants identified as spilling over into barriers to participating in exercise science research.

Institutional and geopolitical factors also shaped unwillingness to participate. Participants described location-dependent safety and avoidance of politically hostile states. Research in TGD health has documented how sociopolitical climates influence research engagement, particularly when discrimination or legal threats are more prevalent (6). Thus, organizational administrators must understand participation in exercise science research cannot be disentangled from broader structural and political contexts.

Notably, several barriers were unrelated to TGD identity and reflected general exercise preferences, such as disinterest in specific exercise modalities or avoidance of strenuous activity. This finding underscores that not all participation barriers described by our sample were unique to TGD individuals. Rather, gender identity interacts with common concerns about time, comfort, and physical effort.

### 4.3 Shared expectations

While many concerns expressed by participants were situated within TGD-specific vulnerabilities, others reflected universal expectations for ethical research conduct. Participants valued clear informed consent, transparency regarding procedures, data security, competent research personnel, and respectful communication. These expectations align with standards codified in human subjects protections and widely endorsed in research ethics literature (33, 35). This convergence suggests that many accommodations viewed by participants as important in a TGD-specific context are in fact expectations of high-quality, participant-centered research design. However, the stakes are amplified for marginalized communities. Historical pathologization and exploitative research practices have contributed to enduring skepticism toward academic institutions (35). Therefore, while ethical best practices apply universally, exercise science researchers must implement them with heightened intentionality and transparency when working with TGD populations.

### 4.4 Structural exclusion and knowledge inequity

The findings suggest that exclusion from exercise science research may be a structural issue of inequity in sport and exercise-related knowledge generation. When TGD individuals disengage from research due to fears of harm, mistrust, or exclusionary research structures, the resulting evidence base may inadequately capture the needs, experiences, and physiological diversity of populations affected by sport, exercise, and physical rehabilitation practices. Consequently, exclusion from research participation may contribute to broader inequities in evidence-informed policy, clinical practice, and sport governance. Fostering equitable participation in exercise science research will require trusted institutions and affirming research environments that demonstrate accountability, transparency, and meaningful protections for TGD participants.

### 4.5 Recommendations for exercise science research

Collectively, the following recommendations reflect a central principle: inclusion of TGD individuals should be the default position in exercise science research. By embedding inclusivity at all levels of the research process, exercise scientists can improve both the ethical integrity and scientific validity of our work. Improving the inclusivity and trustworthiness of exercise science research may support efforts toward reducing health inequities, promoting institutional accountability, and advancing more equitable participation within sport and physical activity systems. Equitable representation of TGD individuals in sport, exercise, and health research may help further broader human rights and public health goals concerning gender equity, reduced inequalities, and inclusive institutions. Institutions, funding agencies, and sport organizations should therefore consider inclusive participant engagement and representative recruitment practices as important components of equitable and ethically responsible evidence generation.

#### 4.5.1 TGD representation in exercise science

Our findings reveal a need to move toward intentional TGD inclusion in exercise science research. Participants consistently emphasized that inclusion must be intentional rather than incidental. Similar to guidance proposed for clinical research (6), exercise scientists should critically evaluate how protocols, eligibility criteria, and analytic plans may explicitly or implicitly exclude TGD individuals. Furthermore, inclusion of TGD individuals should not depend on gender identity as a focal variable. Rather, researchers should include TGD individuals unless there exists a clearly defensible scientific rationale for exclusion. Studies that exclude TGD individuals by default risk perpetuating TGD underrepresentation and limiting the generalizability of findings to the broader population, as systematically excluding even a small percentage of individuals reduces the extent to which results reflect real-world diversity. To help effect this change, institutions, particularly sport and exercise science journals, should adopt policies and guidelines that provide recommendations on best practices and that encourage TGD inclusion in research alongside the cisgender population. Those journals and institutions which currently require the explicit exclusion of TGD or the classification of all research participants based on biological sex should reconsider their positions, particularly within the context of addressing health inequities that arise from physical inactivity.

In our experiences as authors, we can attest that many researchers and editors may argue that the anticipated number of TGD individuals participating in a study limits the ability to draw inferences about them and/or introduces unnecessary variability into analyses. Therefore, their exclusion is always justified. However, these concerns presuppose that (1) a subgroup analysis of only TGD individuals must be performed and (2) that variability between transgender and cisgender individuals exceeds the variability within cisgender individuals to a degree that will negatively affect analysis. These concerns are often unfounded and do not justify exclusion. Methodologically, inclusion does not require subgroup analysis; TGD individuals can be appropriately included in aggregate analyses. As one of our participants noted, sensitivity analysis can reveal if their inclusion affected the results. If so, they may be transparently excluded from the analysis with a described justification. Additionally, concerns about increased variability are not unique to TGD populations and reflect broader challenges inherent to human subjects research, particularly when making generalizable inferences about any minority group (36–38). Excluding TGD individuals perpetuates systemic underrepresentation and reinforces the very absence of data used to rationalize their omission. In our opinion, this intentional omission, and arguments to support it, is an extension of the false characterization of science as apolitical, which leads to reenforcing cisheteronormative culture, shielding oppression, and interpreting transness as incompatible with science (39).

#### 4.5.2 Inclusive data collection and study protocols

Distinguishing sex assigned at birth from gender and avoiding forced binary responses are foundational to participant dignity and accurate representation. This recommendation aligns with clinical research guidance advocating for explicit differentiation of sex and gender in study design and data systems (2, 6). Failure to do so contributes not only to misclassification but to erasure of TGD individuals from datasets, perpetuating underrepresentation. Demographic measures should distinguish sex assigned at birth from gender identity, allow multiple response options, and clearly communicate how these variables will be used analytically.

Most participants in the present study expressed that TGD individuals could be grouped based on their gender (or self-selection) for inclusion in sex-based statistical analysis, noting that hormone replacement therapy (HRT) may significantly alter several characteristics to be more physiologically similar to the sex that better aligns with one's gender. Other participants suggested that anthropometric measurements, hormone profiles, or relevant physiological markers may provide more scientifically defensible analytic groupings than binary sex categories alone. When sex-based classification or exclusion are scientifically necessary (e.g., mechanistic studies of sex-specific biology not understood in the context of HRT), that rationale should be explicitly articulated. Additionally, the SAGER guidelines provide detailed recommendations on how researchers may equitably approach decisions regarding differentiation between sex and gender (40).

Trauma-informed, privacy-protective study procedures were also identified as essential. Little research exists regarding trauma-informed conduct of exercise science research; however, trauma-informed practices are generally characterized by (1) realization that trauma experiences have widespread impact, (2) recognition that specific signs and symptoms may accompany trauma experiences, (3) response from individuals, programs, organizations, and systems to a person experiencing trauma, and (4) re-traumatization should be prevented (41). Most often seen in health care, trauma-informed practices are typically applied by assessing and potentially modifying every aspect of practice to safeguard against re-traumatization (42). Adopting trauma-informed practices in research may begin with implementing participant-centered communication and understanding the basic health effects of trauma experiences across the life span.

Participants stressed minimizing unnecessary bodily exposure and offering flexibility in data collection. Clinical trial guidance similarly recommends adjusting protocols to ensure TGD individuals feel respected and safe while participating (2, 6). Study protocols should be designed to minimize unnecessary gendered assumptions and reduce the risk of dysphoria or stigma. This may include providing private or semi-private testing spaces, offering flexibility in attire requirements, or ensuring that any invasive or body-exposing procedures are justified and transparently explained.

#### 4.5.3 Recruitment

TGD individuals should not have to “decode” whether or not they are eligible to participate in a study. Participants frequently interpreted recruitment materials, researcher communication, and procedural transparency as indicators of whether a research institution could be trusted to engage with TGD individuals safely and respectfully. Recruitment materials should use clear, explicit language that welcomes TGD individuals when appropriate and avoids ambiguous eligibility phrasing, for example, recruiting “queer men” when one really means cisgender, non-heterosexual men. TGD individuals hesitate to participate in research and often do not trust researchers to have their best interests in mind, reflected in the present study and previous research (9, 43). To assuage these concerns, recruitment materials should transparently identify a study's purpose, expectations, the broader benefits to science and society, and the direct benefits to participants. Additionally, monetary compensation appears to strongly influence internal decision-making about research participation, and several of our participants described monetary compensation as a critical facilitator of participation.

Participants' desire for transparent communication parallels ethical recruitment recommendations emphasizing nuanced language, visible inclusion, and collaboration with TGD communities (35). Visible representation, inclusive language, and transparent explanations of study purpose and data use enhance the likelihood of TGD individuals volunteering to participate. Additionally, exercise scientists should prioritize community-mediated recruitment pathways. Inclusive recruitment and communication practices should be viewed as foundational mechanisms through which research institutions communicate legitimacy, accountability, and participant protection.

#### 4.5.4 Community involvement

Importantly, several participants indicated that involvement of the TGD community and TGD stakeholders in a study's design would signal genuine commitment to inclusion. Ethical frameworks for TGD research echo this recommendation, suggesting that peer endorsement and community-based recruitment enhance perceptions of study legitimacy (34, 35). Exercise science researchers should prioritize partnerships with LGBTQIA2S+ organizations to identify the research questions of greatest concern to TGD individuals and effectively plan study protocols and safeguards to investigate those questions. One participant explicitly suggested that exercise scientists wishing to conduct research specifically with TGD individuals should adopt a community-based participatory action research approach to empower TGD communities to effect health and exercise-related outcomes.

#### 4.5.5 Ethical training and protections

Participants expressed that inclusive design alone is insufficient without culturally competent implementation. Organizations should support research teams to pursue training on gender diversity, trauma-informed approaches, respectful communication, and accessibility. Interdisciplinary collaboration with experts in TGD health and physiology may strengthen both methodological rigor and participant safety (2). Furthermore, the responsibility for inclusive and safe exercise science research extends beyond individual researchers to the broader institutions overseeing research practices. Most participants evaluated researcher competence as a proxy for institutional preparedness and legitimacy. Accordingly, research organizations, universities, and laboratories should consider implementing formalized training related to gender diversity, inclusive participant engagement, and trauma-informed research practices for personnel involved in human subjects research in order to ensure that researchers, as institutional representatives, are prepared to carry out inclusive research.

Participants' concerns regarding involuntary disclosure, data misuse, and political targeting further highlight the importance of robust institutional protections surrounding confidentiality and data governance. Institutional review boards should carefully evaluate whether recruitment methods, research procedures, and dissemination strategies adequately minimize risks of reidentification and unintended exposure for TGD individuals, particularly within politically contentious sociocultural environments. The multidimensional nature of safety described by participants suggests that ethical review of exercise science research involving TGD populations may require considerations extending beyond traditional risk assessments. Participants frequently described psychosocial, institutional, and political dimensions of vulnerability that influenced willingness to participate. As such, ethics review processes should consider whether study procedures adequately address risks related to stigma, dysphoria, involuntary disclosure, institutional mistrust, and sociopolitical visibility.

### 4.6 Limitations

Unfortunately, our sample lacks representation from Black participants, who make up ∼13% of TGD adults in the US (44). Two Black individuals scheduled interviews, but subsequently canceled. This likely reflects well-documented structural and intersectional barriers to participation that we failed to overcome rather than a lack of interest in the topic. Black TGD individuals report more frequent and more severe exposure to discrimination, violence, and non-affirmation than White TGD individuals (28). Further, Black communities have a longstanding and well-founded distrust of medical and academic institutions, and Black individuals often avoid research participation due to concerns about exploitation, privacy, and the potential for harm (45). These concerns are likely compounded for Black TGD individuals, who experience intersectional stigma across racialized, gendered, and sexualized axes (26–28). The cumulative effects of racism and transphobia discourage engagement in research spaces, particularly in emerging fields such as exercise science, which may be perceived as predominantly White or cisheteronormative (26, 27). Alongside other structural barriers to participation, Black TGD individuals are underrepresented in digital and community-based LGBTQIA2S+ organizations (5, 45), limiting their exposure to common recruitment pipelines such as our organization-based recruitment approach.

Although participants represented diverse gender identities and geographic regions across the United States, the sample may not fully reflect the breadth of experiences within TGD populations, particularly regarding race, disability, socioeconomic position, athletic involvement, or transition-related experiences. Additionally, as with all human subjects research due to its volunteer nature, participants in this study may have differed in unknown ways from those who did not volunteer. Participants willing to engage in this study may have possessed greater baseline trust in research participation than individuals who declined to participate. However, exploratory subgroup analyses were conducted across several participant demographic characteristics, and these subanalyses did not reveal meaningful inter-group differences or differences relative to the overall sample in category frequency patterns.

### 4.7 Conclusion

The present study contributes to a growing body of literature examining barriers and facilitators to TGD participation in research by situating these factors within exercise science. Willingness to participate is shaped by inclusive design, transparent communication, researcher and institutional trustworthiness, and multidimensional safety appraisal. Many of the facilitating accommodations described align with established ethical standards; however, for TGD individuals, these safeguards are inseparable from lived histories of stigma, exclusion, and vulnerability. Exercise science researchers seeking to promote TGD inclusion must therefore move beyond minimal accommodation toward intentional, affirming, and community-engaged practices that demonstrate trustworthiness in action. This work should include meaningful partnership with TGD people and LGBTQIA2S+ organizations in identifying the most relevant research questions and in shaping the safest, most respectful, and most inclusive methods by which those questions are studied.

More broadly, the present study positions equitable participation in exercise science research as an issue of institutional accountability and governance. Institutional trust, perceptions of legitimacy, and sociopolitical safety shape whether TGD individuals view exercise science research as accessible and safe. Consequently, exclusion from research participation may contribute to inequities in sport-related knowledge generation and limit the applicability of evidence used to guide clinical practice, public health recommendations, and sport policy. Therefore, developing institutionally trustworthy and inclusive research environments may represent an important component of advancing equitable participation within sport, exercise, and health systems.

## Data Availability

The datasets presented in this article are not readily available because due to the small and identifiable nature of the transgender and gender diverse population, participants may be at increased risk of reidentification, even with confidentiality and deidentification procedures. Additionally, interviews included sensitive personal information related to identity, health, and lived experiences. Given these risks, and in light of the Lemkin Institute for Genocide Prevention issuing a warning in January 2026 that the United States may be in the early stages of genocide against transgender and gender diverse individuals, data sharing could pose significant potential harm to participants. Therefore, data will not be made available to protect participant safety and confidentiality. Requests to access the datasets should be directed to Joshua Wooldridge, wooldridgej@moravian.edu.
